# Detection and annotation of plant organs from digitised herbarium scans using deep learning

**DOI:** 10.3897/BDJ.8.e57090

**Published:** 2020-12-10

**Authors:** Sohaib Younis, Marco Schmidt, Claus Weiland, Stefan Dressler, Bernhard Seeger, Thomas Hickler

**Affiliations:** 1 Senckenberg Biodiversity and Climate Research Centre (SBiK-F), Frankfurt am Main, Germany Senckenberg Biodiversity and Climate Research Centre (SBiK-F) Frankfurt am Main Germany; 2 Department of Mathematics and Computer Science, Philipps-University Marburg, Marburg, Germany Department of Mathematics and Computer Science, Philipps-University Marburg Marburg Germany; 3 Palmengarten der Stadt Frankfurt, Frankfurt am Main, Germany Palmengarten der Stadt Frankfurt Frankfurt am Main Germany; 4 Senckenberg Research Institute and Natural History Museum, Frankfurt am Main, Germany Senckenberg Research Institute and Natural History Museum Frankfurt am Main Germany

**Keywords:** herbarium specimens, plant organ detection, deep learning, convolutional neural networks, object detection and localisation, image annotation, digitisation

## Abstract

As herbarium specimens are increasingly becoming digitised and accessible in online repositories, advanced computer vision techniques are being used to extract information from them. The presence of certain plant organs on herbarium sheets is useful information in various scientific contexts and automatic recognition of these organs will help mobilise such information. In our study, we use deep learning to detect plant organs on digitised herbarium specimens with Faster R-CNN. For our experiment, we manually annotated hundreds of herbarium scans with thousands of bounding boxes for six types of plant organs and used them for training and evaluating the plant organ detection model. The model worked particularly well on leaves and stems, while flowers were also present in large numbers in the sheets, but were not equally well recognised.

## Introduction

Herbarium collections have been the basis of systematic botany for centuries. More than 3000 herbaria are active on a global level, comprising ca. 400 million specimens, a number that has doubled since the early 1970s and is growing steadily (Thiers 2020). Accessibility of these collections has been improved by international science infrastructure aggregating specimen data and increasingly also digital images of the specimens. Plant specimens, being usually flat and of a standard format approximating A3 size, are easier to digitise than most other biological collection objects. The Global Plants Initiative (Smith and Figueiredo 2014) has been very successful in digitising type specimens around the world. Single collections, such as the National Museum of Natural History in Paris, have digitised their collections completely (Le Bras et al. 2017) and large scale national or regional digitisation initiatives are already taking place or are planned for the near future (Borsch et al. 2020). Presently, there are more than 27 million plant specimen records with images available via the GBIF platform (www.gbif.org), the vast majority of these images being herbarium scans.

This rising number of digitised herbarium sheets provides an opportunity to employ computer-based image processing techniques, such as deep learning, to automatically identify species and higher taxa (Carranza-Rojas et al. 2017, Younis et al. 2018, Carranza-Rojas et al. 2018) or to extract other useful information from the images, such as the presence of pathogens (as done for live plant photos by Mohanty et al. 2016). Deep learning is a subset of machine learning methods for learning data representation. Deep learning techniques require huge amounts of training data to learn the features and representation of those data for the specified task by fine tuning parameters of hundreds or thousands of neural networks, arranged in multiple layers. Learning the value of these parameters can take vast computer and time resources, especially on huge datasets.

The most common type of deep learning network architecture being used for extracting image features is the Convolutional Neural Network (CNN) (LeCun and Bengio 1995). A convolutional neural network extracts the features of an image by passing through a series of convolutional, non-linear, pooling (image downsampling) layers and passes them to a fully connected layer to obtain the desired output. Each convolutional layer extracts the visual features of the image by applying convolution operations to the image with kernels, using a local receptive field, to produce feature maps and passing it as input to the next layer. The initial layers in the network compute primitive features on the image, such as corners and edges, the deeper layers use these features to compute more complex features consisting of curves and basic shapes and the deepest layers combine these shapes and curves to create recognisable shapes of the concepts in the image (Yosinski et al. 2014, Zeiler and Fergus 2014).

In this paper, we use deep learning for detecting plant organs on herbarium scans. The plant organs are detected using an object detection network, which works by localising each object with a bounding box on the image and classifying it. There are many types of networks, based on CNN, used for this application. In this study, a network called Faster R-CNN (Ren et al. 2015) was used, which is part of the R-CNN family for object detection. Region-based Convolutional Networks (R-CNN) identify objects and their locations in an image. Faster R-CNN networks have shown state-of-the-art performances in various object detection applications and competitions (Zhao et al. 2019). Therefore, many researchers have explored the use of CNN and particularly Faster R-CNN for detecting various plant organs, such as flowers, fruits and seedlings (Sa et al. 2016, Stein et al. 2016, Häni et al. 2020, Mai et al. 2018, Sun et al. 2018, Bargoti and Underwood 2017, Jiang et al. 2019, Ott et al. 2020, Weaver et al. 2020). To our knowledge, this is the first time object detection has been used to detect both vegetative and reproductive plant organs on herbarium scans. Identifying and localising plant organs on herbarium sheets is a first necessary step for some interesting applications. The presence and state of organs, such as leaves, flowers and fruits, can be used in phenological studies over long time periods and may give us more insight into climate change effects since the time of the Industrial Revolution (Willis et al. 2017, Lang et al. 2019).

## Methods

### Network architecture

A typical object detection network consists of object localisation and classification integrated into one convolutional network. There are two main types of meta-architectures available for this application: single stage detectors like Single Shot Multibox Detectors (SSD) (Liu et al. 2016) and 'You only look once' (YOLO) (Redmon et al. 2016) and two-stage, region-based CNN detectors, such as Faster R-CNN. Single stage detectors use a single feed-forward network to predict object class probabilities along with bounding box coordinates on the image. Faster R-CNN is composed of three modules: 1) a deep CNN image feature extraction network, 2) a Region Proposal Network (RPN), used for detection of a predefined number of Regions of Interests (RoIs) where the object(s) of interest could reside within the image, followed by 3) Fast R-CNN (Girshick 2015), computes a classification score along with class-specific bounding box regression for each of these regions. The main reason for choosing Faster R-CNN for organ detection is because it is generally more accurate, particularly for large and small objects, than single stage detectors like SSD when speed and memory consumption are not as important as overall accuracy (Huang et al. 2017).

**Figure 1.**
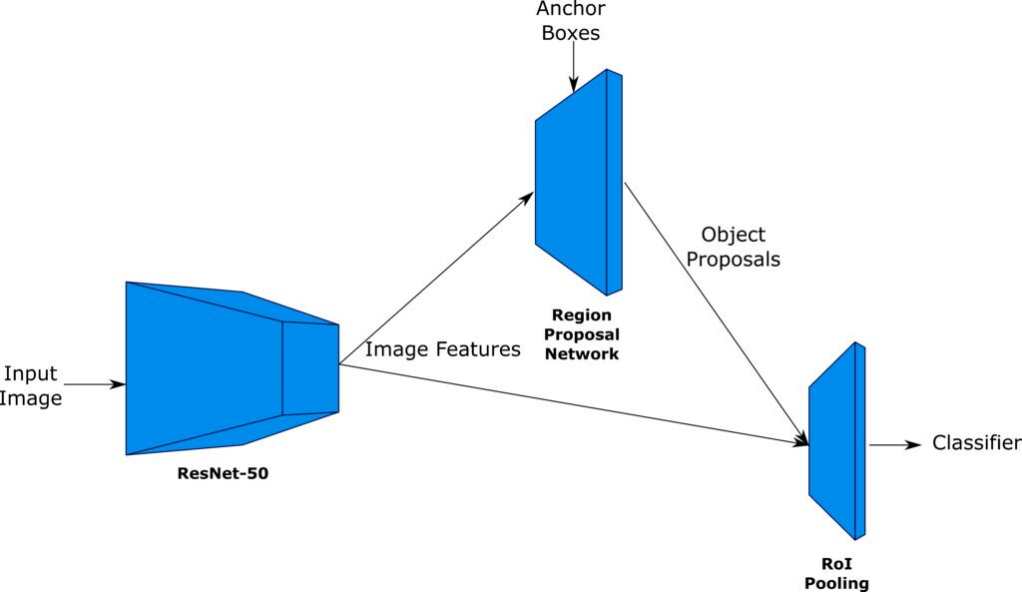
An illustration of the Faster R-CNN architecture, with ResNet for image feature extraction, RPN for generating object proposals and RoI Pooling for creating fixed-size feature maps for each proposal.

The CNN feature extraction network used in this paper is based on the ResNet-50 architecture (He et al. 2016), without the final fully-connected layer. The Region Proposal Network (RPN) creates thousands of prior or anchor boxes to estimate the location of objects in the image. The anchor boxes are predefined bounding boxes of certain height and width tiled across the image, determined by their scale and aspect ratios, in order to capture different sizes of objects of specific classes. The RPN generates these proposals by adjusting these anchors with coordinate offsets of the object bounding boxes and predicts the possibility of each anchor being a foreground object or a background. These proposals are sorted according to their score and top N proposals are selected by Non-Maximum Suppression (NMS), which are then passed to Fast R-CNN stage. NMS reduces the high number of proposals for the next stage by short-listing the proposals with the highest score having minimum overlap with each other by removing the proposals with overlap above a predefined threshold for each category. In the next stage, the proposals with feature maps of different shapes are pooled with a ROI pooling layer, which performs max-pooling on the inputs of non-uniform sizes to obtain a fixed number of uniform size feature maps. These feature maps are propagated through fully-connected layers, which end in two siblings fully-connected layers for object classification and bounding box regression, respectively. An illustration of Faster R-CNN is shown in Fig. 1.

### Image Annotation

**Figure 2.**
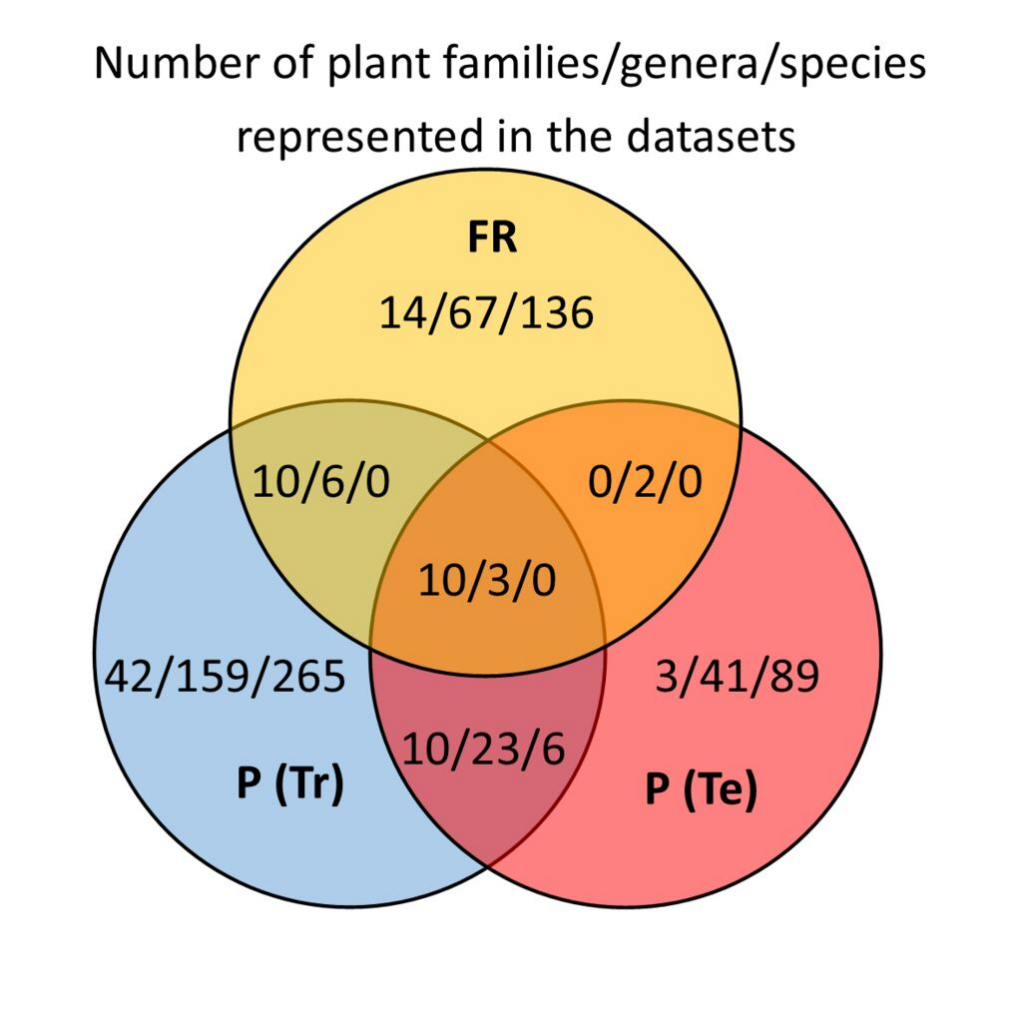
Number of taxa of different rank for the three datasets with overlaps at family, genus and species level. P(Tr), P(Te): MNHN Paris Herbarium training and test datasets, FR: Herbarium Senckenbergianum dataset.

**Figure 3.**
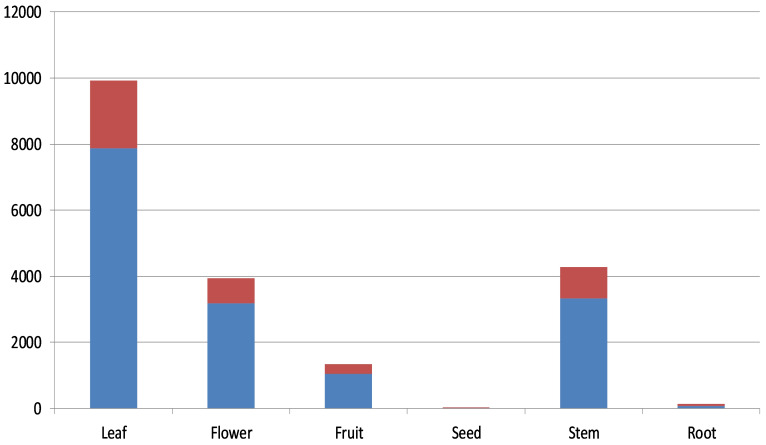
A column chart showing the number of annotated bounding boxes for each organ. Red: Test subset, Blue: Training subset.

**Figure 4.**
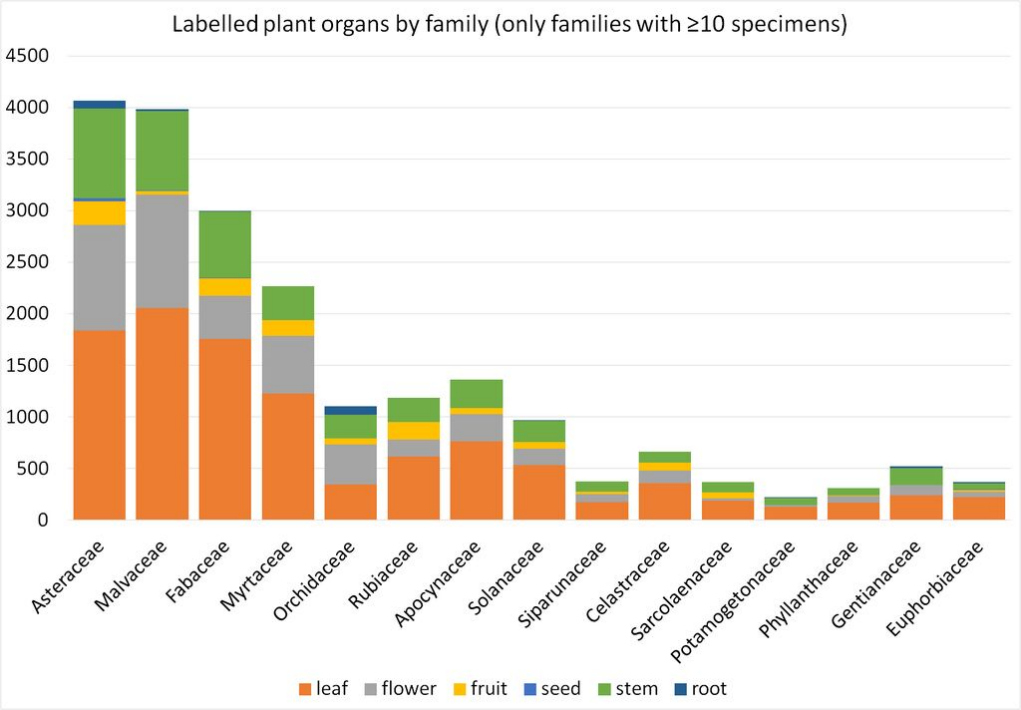
Families of labelled specimens (ordered by number of specimens) with number of labelled plant organs. The share of the plant organs differs between families, which may be due to factors depending on the plant itself and collecting habits (season, selection of identifiable specimens).

The herbarium scans annotated for training the object detection network were selected from the MNHN (Muséum national d’Histoire naturelle) vascular plant herbarium collection dataset in Paris (Le Bras et al. 2017), from open access images contributed to the GBIF portal (MNHN and Chagnoux 2020). A total of 653 images were downloaded and rescaled from their original average size of ca. 5100 by 3500 pixels to 1200 by 800 pixels, in order to preserve the aspect ratio of the scans and to speed up the learning by reducing the number of pixels. The images were selected manually from a large collection of scans, having minimum visual overlap between organs, while covering a broad range of taxa and morphology (Fig. 2, Suppl. material 2). All these images were annotated for six different types of organs (Suppl. material 1) using LabelImg (Tzutalin 2015), a Python graphical toolkit for image annotation using bounding boxes. The average rate for manual image annotation was 8 to 15 herbarium sheets per hour, depending on the difficulty and number of bounding boxes to be annotated. The total number of annotated bounding boxes for all 653 images was 19654, with an average of 30.1 bounding boxes per image. From these 653 annotated images, 155 of them were either annotated or verified by an expert, making a validated subset hence used for testing and the 498 were used for training, as shown in Fig. 3 and Fig. 4 and in more detail in Table 1.

Preparing our data was not always straight-forward. The manual localisation and labelling of plant organs from specimens encountered the following difficulties: buds, flowers and fruits are different stages emerging in the life cycle of plant reproductive organs and, in some cases, it was therefore difficult to find a clear distinction between these structures. In some taxa, different plant organs were impossible to separate as these were small and crowded, for example, in dense inflorescences with bracts and flowers or stems densely covered by leaves. In a few cases, it was also hard to differentiate from the digital image between roots and stolons or other stem structures. In all of these cases, we placed our labelled boxes in a way to best characterise the respective plant organ. Sometimes, this involved including parts of other organs and, at other times, if sufficient clearly assignable material were available, difficult parts were left out.

### Implementation

**Figure 5.**
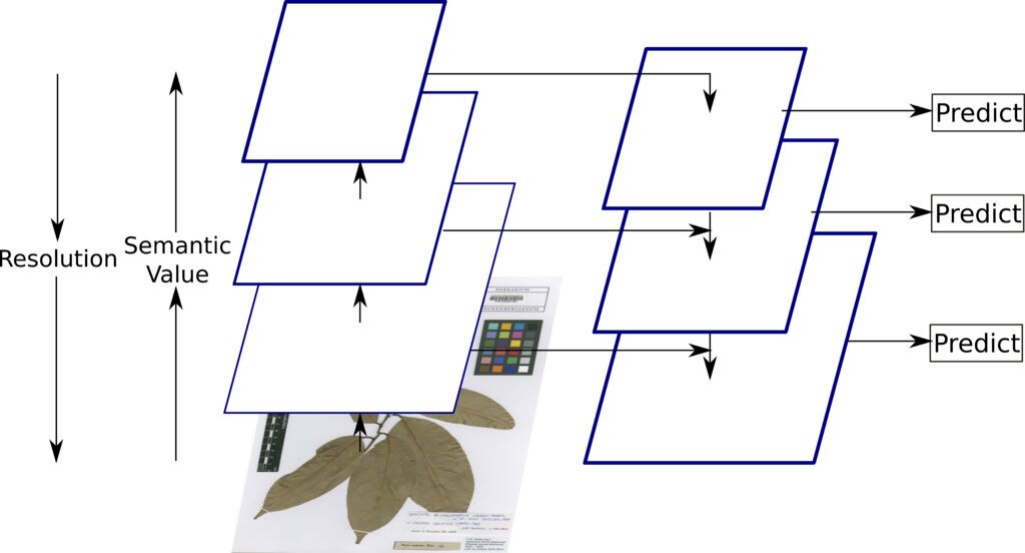
An illustration of Feature Pyramid Network, where feature maps are indicated by blue outlines and thicker outlines denote semantically stronger features (Lin et al. 2017).

The object recognition task was performed using Faster R-CNN, as described in the network architecture, with the Feature Pyramid Network (Lin et al. 2017) backbone. The Feature Pyramid Network increases the accuracy of the object detection task by generating multi-scale feature maps from a single scale feature map of ResNet output, by making top-down pathways in addition to the usual bottom-up pathways used by a regular convolutional network for feature extraction, where each layer of the network represents one pyramid level. The bottom–up pathway increases the semantic value of the image features, from corners and edges in the initial layers to detecting high level structures and shapes of objects in the image in the final layers, while reducing its resolution at each layer. The top-down pathway then reconstructs higher resolution layers from the most semantically rich layer, with predictions made independently at all levels as shown in Fig. 5. This approach provides Faster R-CNN with feature maps at different resolutions for detecting objects of multiple scales.

In order to reduce the training time and, more importantly, because of the small size of the training dataset, transfer learning (Yosinski et al. 2014) was implemented to initialise the model weights pre-trained on the ImageNet dataset (Deng et al. 2009). Since the initial layers of a CNN usually learn very generic features that can also be used in new contexts, pre-trained weights can initialise the weights for these layers. For the deeper layers, transfer learning is used to initialise the parameter weights pre-trained on the ImageNet dataset and then fine-tuned during training, using the annotated herbarium scan dataset until convergence.

The model was implemented with the Detectron2 (Wu et al. 2019) library in PyTorch framework and trained using Stochastic Gradient Descent optimiser with a learning rate of 0.0025 and momentum of 0.9. The anchor generator in the Region Proposal Network (see section above on network architecture) had six anchor scales [32, 64, 128, 256, 512, 1024] (square root of area in absolute pixels) each with three aspect ratios of [1:2, 1:1, 2:1]. The thresholds for non-maximum suppression (NMS) were 0.6 for training and 0.25 for testing, respectively.

Due to the large image size and additional parameters of Faster R-CNN, a minibatch size of four images per GPU (TITAN Xp) was selected for training the model. The model was trained twice, once with a training subset of 498 images on a single GPU for 9000 iterations and performance evaluated on the test subset of 155 images, also on a single GPU and then trained again on all 653 annotated images on three GPUs for 18000 iterations for predicting plant organs on another un-annotated independent dataset to evaluate our method. This dataset consists of 708 full scale herbarium scans, with an average size of ca. 9600 by 6500 pixels, from the Herbarium Senckenbergianum (FR) (Otte et al. 2011) with a different set of species (Fig. 2) and geographical origins, which is also available at GBIF (Senckenberg 2020). The Python code and the trained model have been made available at GitHub (Younis 2020).

## Results

The predictions of the organ detection model provides a list of bounding boxes for each organ, along with the confidence levels and their class labels. The performance of the model was evaluated using the COCO evaluation metric (Lin et al. 2014), which determines whether the predicted organs and their locations are correct. The minimum threshold chosen for any prediction to be acceptable is having a confidence score (probability) of 0.5. The COCO method calculates average precision (with values from 0 to 100), which is a metric that encapsulates both precision and recall of the detection, for the entire predictions and each class of organs at different levels of Intersection over Union (IoU). IoU is an evaluation metric that quantifies the overlap of the predicted bounding boxes with the ground-truth bounding boxes. The IoU score ranges from 0 to 1, the higher the overlap, the higher the IoU score. The evaluation method considers all predictions as positive that have IoU of at least 0.5 and the average precision at this level of IoU is called AP50. Similarly, the average precision with a minimum IoU of 0.75 is called AP75, whereas AP is the average over 10 IoU levels from 0.5 to 0.95 with a step size of 0.05. The precision metrics evaluated on the predicted organs on the test subset are shown in Table 2. The COCO method also calculates the AP for each category, as shown in Table 3, along with the total bounding boxes for each category in the test subset.

From the predicted annotations of the model for plant organs on 708 full scale herbarium scans from the Herbarium Senckenbergianum dataset, trained on the 653 annotated MNHN Paris Herbarium dataset, 203 were manually verified and corrected to evaluate the predictions. The organ detection model was successfully able to detect almost all plant organs in the majority of scans, as shown by the images in Fig. 6. The dataset of these 203 herbarium scans, along with the result of detections and the annotations, is available at PANGAEA Younis et al. 2020.

The performance of the model on the verified annotated Herbarium Senckenbergianum dataset is shown in Table 4 and Table 5. The average precision on these 203 scans is generally higher than the MNHN Paris Herbarium test subset, there being two main reason for this: 1) The organ detection model for full scale detection was trained on all 653 images of the MNHN Paris Herbarium annotated dataset before detection on the Herbarium Senckenbergianum dataset, 2) The annotation of these 203 images from the Herbarium Senckenbergianum dataset were done, based on the predictions of organs on scans as shown in Fig. 6.

## Discussion

This paper presents a method to detect multiple types of plant organs on herbarium scans. For this research, we annotated hundreds of images with thousands of bounding boxes by hand for each possible plant organ. A subset of these annotated scans was then used for training of deep learning for organ detection. After training, the model was used to predict the type and location of plant organs on the test subset. The automated detection of plant organs in our study was most successful for leaves and stems (Table 3 and Table 5). Best AP values for leaves are likely due to the largest set of annotated bounding boxes. Good values for stems and roots may be explained by the relative uniformity of these organs throughout the plant kingdom, as compared to the morphologically more diverse flowers and fruits in between these. Seeds are rarely visible on herbarium sheets and require more training material.

The model was trained again on all the annotated scans earlier and tested on a different un-annotated dataset. The model performed well, based on visual inspection. In order to evaluate the performance of the model with an average precision metric, around 200 of these scans were annotated by hand, based on the predicted bounding boxes. The predicted bounding boxes dramatically reduced the time to annotate these scans, since the predictions for leaves and stems were fairly accurate. After being annotated, these scans were compared with the predictions to evaluate the precision of the organ detection model on this dataset.

We consider our study as a 'real-life' pioneer study with inherent biases. The training and test datasets from MNHN Paris Herbarium are from the same collection, while the Herbarium Senckenbergianum specimens are from an independent collection with different geographical and taxonomic focus, but still with a number of higher taxa in common with MNHN Paris Herbarium. The different datasets overlap mainly on the family level, partly on genus level and only slightly between the MNHN Paris Herbarium training and test datasets at species level (Fig. 2, Suppl. material 2). Therefore, we can exclude organ recognition being based upon species-specific features. As in nature itself and the collections represented here, families are not represented equally. Likewise, the number of labelled organs, represented in our dataset, is far from balanced and biased both by the natural distribution of these organs in the sampled taxa and by the selection of material by the collectors. Roots, for example, are mainly represented in Asteraceae and Orchidaceae, families with many small and herbaceous species (Fig. 4, Suppl. material 3). In order to better understand the difference in average precision of organ detection across different taxa, further studies are necessary. A promising strategy would be to employ data augmentation to create artificially-balanced distributions of organs and taxa (Shorten and Khoshgoftaar 2019). The current study focuses on the analysis and the provision of annotated datasets of actual herbarium specimens, involving the aforementioned constraints rooted in the morphology of the specimens concerned and not simulated data. It would also be interesting to compare a general organ recognition with taxon-specific approaches. Especially for fruits and flowers, we have very different shapes between taxa and also the possible distinction between different developmental stages depends a lot on the taxon.

Most computer vision approaches on plants focus on live plants, often in the context of agriculture or plant breeding and, therefore, include only a limited set of taxa. The present approach not only targets a much larger group of organisms and morphological diversity, comparable to applications in citizen science (Wäldchen and Mäder 2019), but can also be applied on a wider time-scale by including collection objects from hundreds of years of botanical research. Some significant recent similar approaches to detect plant organs on herbarium scans are GinJinn (Ott et al. 2020) and LeafMachine (Weaver et al. 2020). GinJinn uses an object-detection pipeline for automated feature extraction from herbarium specimens. This pipeline can be used to detect any type of plant organ, which the authors of this research demonstrated by detecting leaves on a sample dataset. LeafMachine is another approach which tries to automate extraction of leaf traits, such as class, size and number, from digitised herbarium specimens with machine learning.

## Conclusions

Our present work focuses on the detection of plant organs from specimen images. The presence of flowers and fruits on specimens is a new source of data for phenological studies (Willis et al. 2017), interesting in the context of climate change. Presence of roots would identify plant specimens potentially containing root symbionts, such as mycorrhizal fungi or N-fixing bacteria, for further study by microbiological or genetic methods (Heberling and Burke 2019). Up to now, this requires visual examination of the specimens by humans; however, an automated approach using computer vision would considerably reduce the effort. Furthermore, the detection and localisation of specific plant organs on a herbarium sheet would also enable or improve further computer-vision applications, including quantitative approaches, based on counting these organs, improved recognition of qualitative organ-specific traits, such as leaf shape, as well as quantitative measures, such as leaf area or fruit size.

Localisation of plant organs will improve automated recognition and measurements of organ-specific traits, by preselecting appropriate training material for these approaches. The general approach of measuring traits from images instead of the specimen itself has been shown to be precise, except for very small objects (Borges et al. 2020). Of course, measurements that involve further processing of plant parts, as often done in traditional morphological studies on herbarium specimens, are not possible from images.

Automated pathogen detection on collection material will also profit from the segmentation of plant organs from Herbarium sheet images, as many pathogens or symptoms of a plant disease only occur on specific organs. Studies on gall midges (Veenstra 2012) have found herbarium specimens to be interesting study objects and would potentially profit from computer vision.

Manual annotation of herbarium specimens with bounding boxes, as done for the training and test datasets in this study, is a rather time-consuming process. Verification and correction of automatically-annotated specimens is considerably faster, especially if the error rate is low. By iteratively incorporating expert-verified computer-generated data into new training datasets, the results can be further improved with reasonable efforts using Continual Learning (Parisi et al. 2019).

## Supplementary Material

722AB184-5B12-5547-967E-6D3A34D2D5C8 10.3897/BDJ.8.e57090.suppl1 Supplementary material 1 Plant Organ Annotations Data type XML Files Brief description The zip archive provides annotations for both Herbarium Senckenbergianum and MNHN Paris Herbarium datasets. File: oo_472998.zip https://binary.pensoft.net/file/472998 Sohaib Younis, Marco Schmidt, Claus Weiland

37DD5C1D-8805-590E-A421-4AAC440A3269 10.3897/BDJ.8.e57090.suppl2 Supplementary material 2 Specimen List Data type CSV File Brief description The file provides a list for all the specimens, showing their taxonomy, organ count and URLs. File: oo_473000.csv https://binary.pensoft.net/file/473000 Sohaib Younis, Marco Schmidt

B9F9B259-2AA7-5E04-87EC-0E4D6DE417B4 10.3897/BDJ.8.e57090.suppl3 Supplementary material 3 Family organ count Data type CSV File Brief description The file provides a list of the total annotated organs for each family. File: oo_473004.csv https://binary.pensoft.net/file/473004 Sohaib Younis, Marco Schmidt

## Figures and Tables

**Figure 6a.**
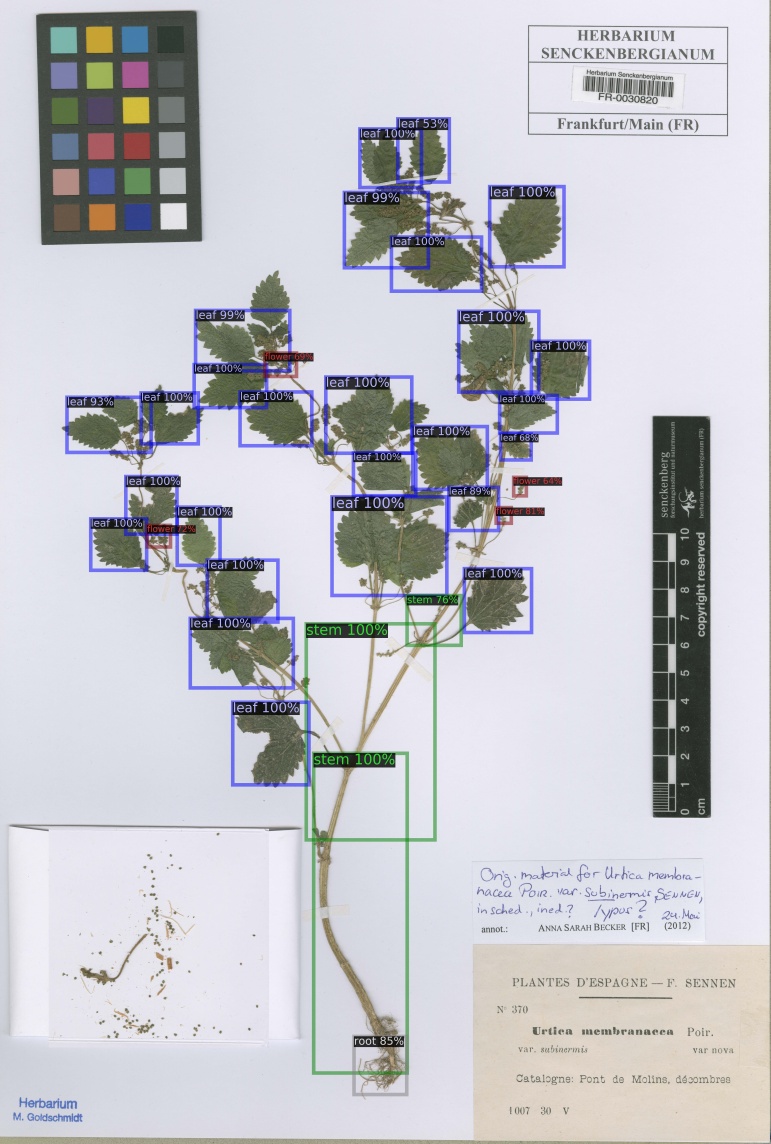


**Figure 6b.**
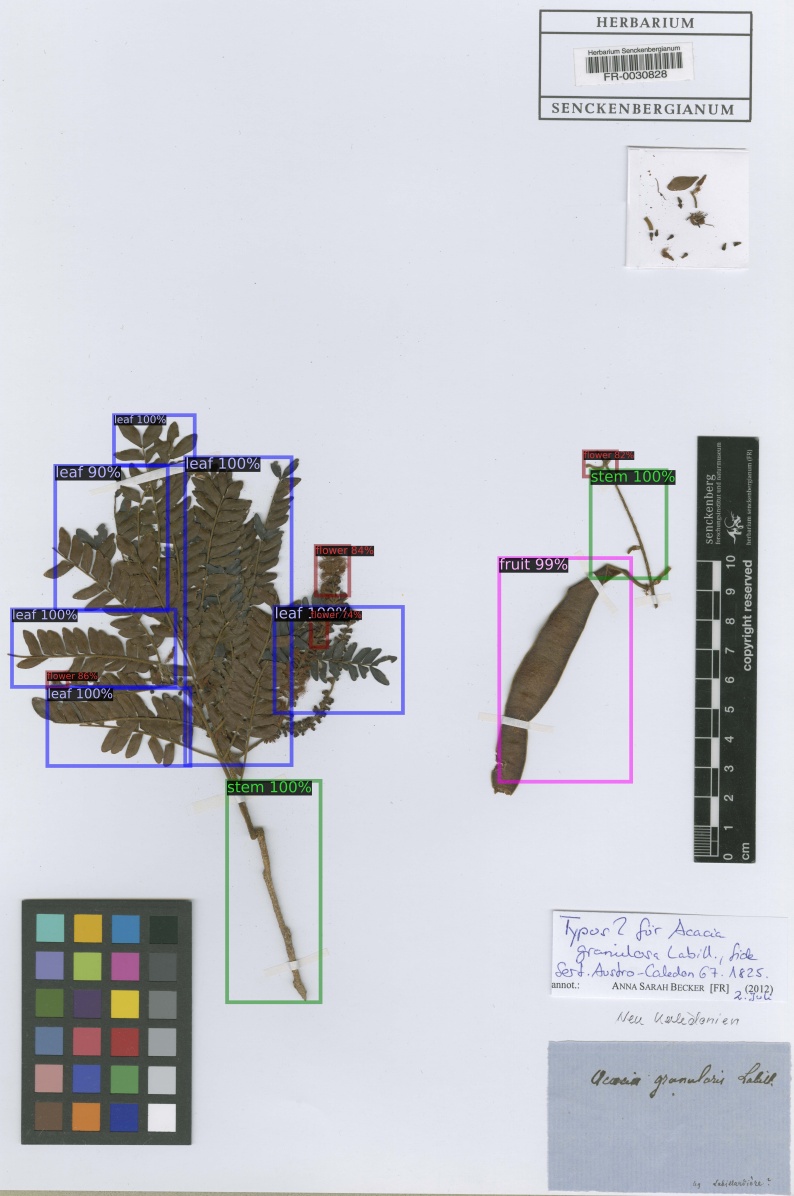


**Figure 6c.**
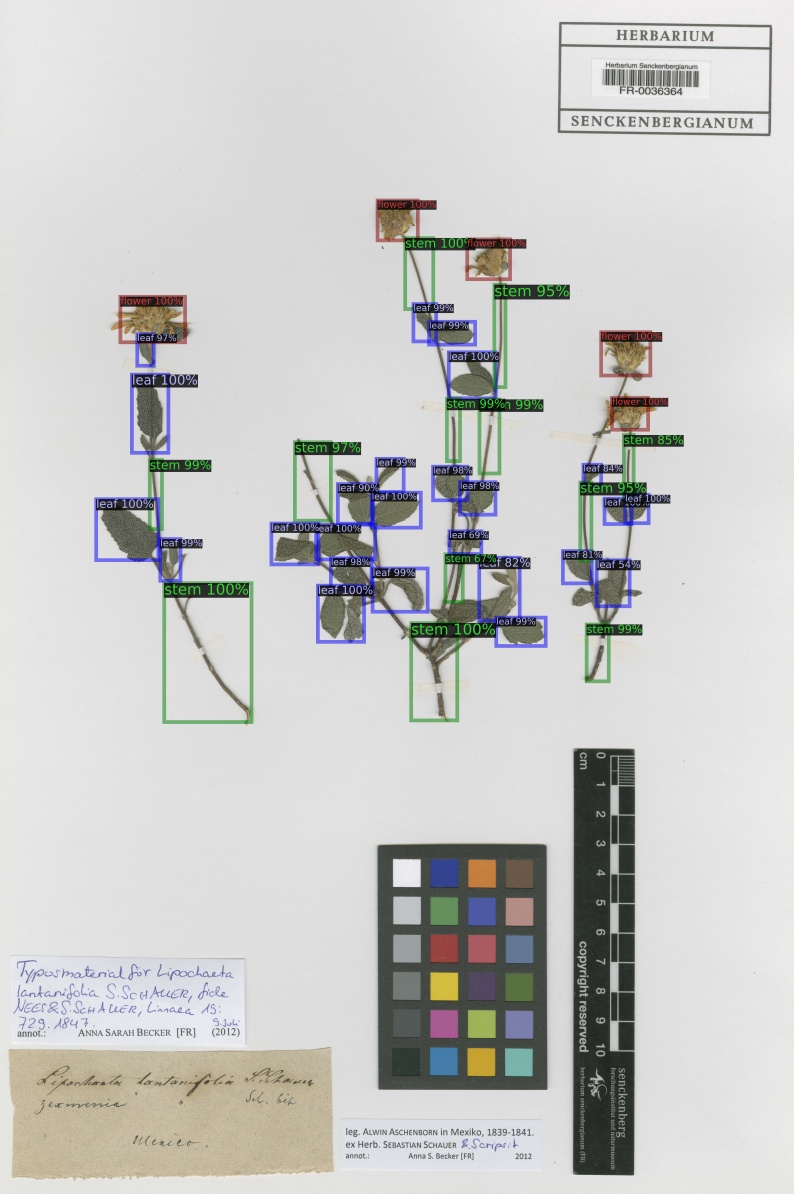


**Figure 6d.**
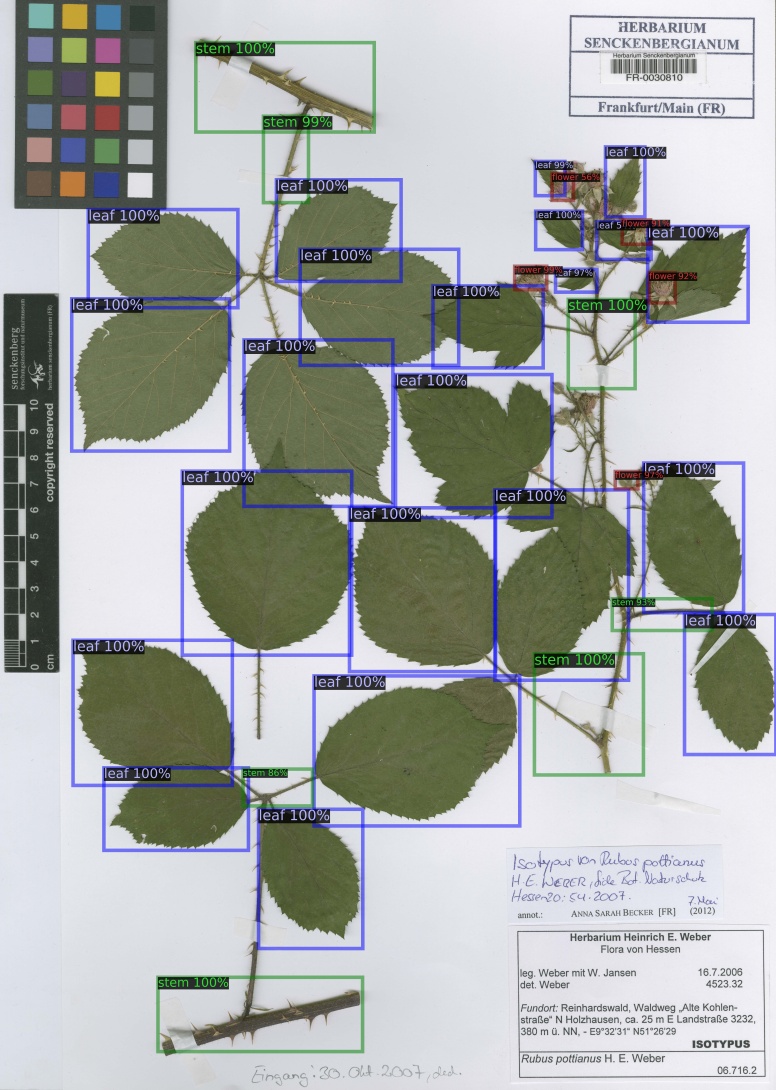


**Table 1.** The number of annotated bounding boxes for each plant organ in training and test subset.

Category	Training subset (498 images)	Test subset (155 images)	Complete dataset (653 images)
Leaf	7886	2051	9937
Flower	3179	763	3942
Fruit	1047	296	1343
Seed	4	6	10
Stem	3323	961	4284
Root	78	60	138
Total	15517	4137	19654

**Table 2.** The precision of the predictions on the MNHN Paris Herbarium test subset with COCO evaluation method.

AP50	AP75	AP
22.8	6.8	9.7

**Table 3.** Average Precision of each type of organ along with the total bounding boxes for each category in the test subset.

Category	Bounding Boxes	AP
Leaf	2051	26.5
Flower	763	4.7
Fruit	296	7.8
Seed	6	0.0
Stem	961	9.9
Root	60	9.4

**Table 4.** Result of model evaluation on the Herbarium Senckenbergianum annotated dataset.

AP50	AP75	AP
32.1	16.1	16.8

**Table 5.** Average Precision of each type of organ along with the total bounding boxes for each category in the Herbarium Senckenbergianum annotated dataset.

Category	Bounding Boxes	AP
Leaf	3362	37.9
Flower	1921	18.3
Fruit	183	7.9
Seed	47	0.0
Stem	1063	25.1
Root	117	11.8
